# The immunogenicity database collaborative: a standardized, publicly available database for clinical immunogenicity observations and insights

**DOI:** 10.3389/fimmu.2026.1816949

**Published:** 2026-06-30

**Authors:** Sudhanshu Agnihotri, Bruno Gonzalez-Nolasco, Brinda Monian, Sofie Pattijn, Chloé Ackaert, Patrick Wu, Hubert Kettenberger, Sophie Tourdot, Timothy Hickling, Zicheng Hu, Richard E. Higgs, Daniel S. Leventhal

**Affiliations:** 1 Department of Pharmaceutical Sciences, University at Buffalo, The State University of New York, Buffalo, NY, United States; 2 Early Development Services, Lonza Biologics Inc., Cambridge, MA, United States; 3 Generate Biomedicines, Somerville, MA, United States; 4 In Vitro Immunology, IQVIA Laboratories, Gosselies, Belgium; 5 Department of Translational Pharmacokinetics and Pharmacodynamics, Genentech Inc, South San Francisco, CA, United States; 6 Large Molecule Research, Roche Pharma Research and Early Development, Roche Innovation Center Munich, Penzberg, Germany; 7 Pharmacokinetics, Dynamics and Metabolism, Pfizer Inc., Andover, MA, United States; 8 Pharma Research and Early Development, Roche Innovation Centre Welwyn, Roche, Welwyn Garden City, United Kingdom; 9 Quasor Ltd., Loughborough, England, United Kingdom; 10 Eli Lilly and Company, Indianapolis, IN, United States; 11 Xaira Therapeutics, South San Francisco, CA, United States; 12 Tactyl LLC, St. Petersburg, FL, United States

**Keywords:** anti-drug antibodies, biologics, database, immunogenicity, immunogenicity risk assessment

## Abstract

Anti-drug antibodies (ADAs) against biotherapeutics remain difficult to predict, limiting efforts to assess and mitigate immunogenicity risk prior to clinical development. Existing immunogenicity data are fragmented across disparate sources and reported using inconsistent definitions, creating a major barrier to understanding the drivers of ADA formation. To address this challenge, we established the Immunogenicity Database Collaborative (IDC), launched its public website (https://www.immunogenicitydb.org), and developed the first release of the Immunogenicity Dataset (IDC DS V1), a structured clinical immunogenicity dataset integrating therapeutic characteristics, amino acid sequence information, and patient cohort-level clinical data curated from publicly available sources. The IDC DS V1 contains 4,146 ADA-related datapoints spanning 1,788 cohorts, 727 clinical trials, and 218 therapeutics. Analysis of the dataset highlights trends in ADA frequency, reveals important sources of variability across clinical contexts, and identifies key factors associated with immunogenicity risk. The IDC provides a foundational resource to standardize clinical immunogenicity data and support immunogenicity risk assessment across the biopharmaceutical industry. In addition to the current dataset release, it establishes an extensible data architecture and framework for future community-driven expansion into additional areas of immunogenicity research.

## 1 Introduction

The inclusion of biologics into modern medicine has revolutionized the treatment of human disease. However, the therapeutic potential of protein-based drugs can be undermined by unwanted immunogenicity, where the immune system generates anti-drug antibodies (ADAs) against the biologic (1, 2). ADAs can neutralize drug activity (3), accelerate clearance (4), or cause hypersensitivity reactions (5), ultimately compromising safety and efficacy (6–11). In some cases, immunogenicity detected late in clinical development can halt otherwise promising therapeutics (12). These risks underscore the importance of deepening our understanding of the root causes of immunogenicity, as well as continually advancing preclinical risk assessment tools and mitigation strategies.

Immunogenicity risk is influenced by multiple factors that can be broadly categorized into product-, treatment-, and patient-related variables (13, 14). Many product-intrinsic risks can be assessed preclinically, including a drug’s mechanism of action (MOA), antigenicity (e.g., T and B cell epitope content), developability attributes (e.g., aggregation propensity, polyreactivity, etc.), and drug product critical quality attributes (CQAs) (e.g., impurity content, host-cell protein levels, etc.) (15). Treatment-related factors include a drug’s dose quantity, dose frequency and route of administration (ROA), while patient-related factors include immune status, concomitant medications, and genetic background (16). The complex interplay between these variables can further impact ADA outcomes.

While these factors are well established as independent contributors, their complex, context-dependent interplay with ADA outcomes makes it challenging to deconvolute their relative contributions to immunogenicity risk. For example, a non-human, highly antigenic protein may not trigger ADA formation in an immunosuppressed population, while a fully humanized therapeutic may elicit strong responses if its target is expressed on antigen-presenting cells (16, 17). A wide range of clinical and ADA assay technical factors can influence reported immunogenicity outcomes, therefore regulatory guidance has historically discouraged direct comparison of ADA rates across therapeutics (18). Nonetheless, when scale meets sufficient methodological and clinical context, such analyses can yield valuable scientific insights, and thus high-quality, fit-for-purpose datasets that link immunogenicity outcomes to relevant risk factors are of great value to the field.

Prior publicly available clinical immunogenicity data are fragmented across FDA labels, ClinicalTrials.gov entries, EMA reports, and throughout primary literature. It often contains inconsistent reporting of ADA outcomes (e.g., transient ADA, treatment-emergent ADA, etc.) and limited methodological details for ADA assessments. Published aggregated datasets utilized in the field frequently report a single ADA frequency and often differing values for the same molecules (**Supplementary Figure S1**) (19–22). This heterogeneity impairs data accessibility, hinders context aware comparisons and limits the ability to elucidate drivers of immunogenicity risk.

To address these gaps, we established the Immunogenicity Database Collaborative (IDC) and developed the IDC Database (IDC DB), a relational data framework designed to integrate immunogenicity observations with relevant contextual metadata, including therapeutic properties, amino acid sequence, and clinical context. The first public release of the IDC DB is the IDC Dataset V1 (IDC DS V1), a curated and standardized clinical immunogenicity dataset accompanied by supporting documentation. The IDC DB is intended to serve a broad range of stakeholders, including immunogenicity scientists, protein engineers, translational scientists, data scientists, regulatory scientists, and developers of predictive immunogenicity risk assessment methodologies. Potential applications include identifying factors associated with ADA formation, benchmarking immunogenicity risk assessment approaches, supporting development of future machine learning models, and improving our understanding of the clinical relevance of immunogenicity. Here, we describe the dataset architecture, characterize its contents, and demonstrate its utility by exploring variable contributions to ADA frequency across biologics and a broad range of clinical contexts.

## 2 Materials and methods

### 2.1 IDC sources and selection of initial set of therapeutics

The IDC DS V1 was constructed through domain expert-led systematic extraction and curation of publicly available information on clinical immunogenicity. Development of the database structure, and subsequent construction and auditing of the IDC DS V1 included expertise spanning immunobiology, clinical immunogenicity, bioanalytics, toxicology, bioinformatics, data architecture, machine learning, and biologics development. Contributors included active academic and industry scientists with experience in immunogenicity risk assessment, enabling both the design of a fit-for-purpose data structure and the interpretation and harmonization of heterogeneous immunogenicity data sources. Primary data sources included U.S. Food and Drug Administration (FDA) (https://nctr-crs.fda.gov/fdalabel/ui/search) and European Medicines Agency (EMA) product labels (www.ema.europa.eu/en/search), ClinicalTrials.gov, the EU Clinical Trials Register (www.clinicaltrialsregister.eu), WHO International Nonproprietary Name (INN) databases (www.who.int/teams/health-product-and-policy-standards/inn), and curated repositories such as DrugBank (23) and Thera-SAbDab (24), supplemented by peer-reviewed publications, patent filings, and regulatory submissions where available. Data were organized into three interlinked tables, Therapeutics, Sequences, and Clinical Trials, each connected by unique identifiers to enable relational mapping.

IDC DS V1 was not intended to represent an exhaustive collection of all published clinical immunogenicity data. Rather, the initial objective was to develop and validate a relational database structure capable of capturing immunogenicity observations and associated contextual metadata across diverse therapeutic and clinical settings. To demonstrate the utility of this framework, an initial set of therapeutics was prioritized for manual curation, with emphasis placed on monoclonal antibodies due to the relative abundance of publicly available clinical immunogenicity data for this therapeutic modality. Additional biologic modalities were included to ensure the data structure could accommodate diverse therapeutic formats.

This initial set of therapeutics were selected to provide a broad range of reported ADA frequencies and clinically relevant contexts, including examples of biosimilars, immunomodulatory co-medications, diverse mechanisms of action, multiple disease indications, and varied routes of administration. Clinical data were primarily sourced from ClinicalTrials.gov and the European Union Clinical Trials Registry through searches using International Nonproprietary Names (INNs) and common product names. Only studies reporting immunogenicity-related outcomes (e.g., ADA frequency, neutralizing antibody frequency, ADA titers, or related measures) were incorporated into the dataset. To a lesser extent clinical data and metadata were obtained from FDA labels, review articles, and primary literature identified through targeted searches, recommendations from IDC contributors, or manual review performed during dataset development. Please refer to the “Trial External Source” and “External Source Identifier” columns within the Clinical trial table for data source information.

### 2.2 Sections of IDC DS V1

The Therapeutics table (example shown in **Supplementary Table 1**) captured drug-level attributes, including INN, trade names, antibody backbone (e.g., IgG1, IgG4, Fc-fusion), species origin (murine, chimeric, humanized, or human), expression system, disease indication, molecular target, and mechanism of action. Mechanistic characteristics were recorded under the “Mechanism of Action” column to highlight drugs that are directly immunomodulatory, such as immune system activators that confer heightened risk in certain patient populations. Regulatory milestones were also documented, including FDA and EMA approval status and timelines when obtainable. Each therapeutic was assigned a unique Therapeutic_ID that served as the primary key across tables. Biosimilars were annotated in the field “Labelled as Biosimilar?” and linked to their parent molecule through the “Progeny of” field, while sequence cross-references were captured through Sequence IDC Identifiers.

The Sequences table (example shown **Supplementary Table 2**) recorded amino acid sequences for therapeutic agents, organized by heavy and light chains and linked to their parent therapeutic via Therapeutic_ID. Each sequence was annotated with chain type, sequence source, and domain identity, and curated primarily from IMGT (25), regulatory filings, and patent databases, with additional references from repositories such as the RCSB Protein Data Bank (26). For biosimilars, only the parent drug sequence was reported since by definition the primary amino acid sequences should be identical. A limitation of this table is that amino acid sequences for certain novel constructs or investigational biologics were not publicly available.

The Clinical Trials table (example shown in **Supplementary Table 3**) formed the core of the dataset, capturing immunogenicity outcomes at the cohort level within each trial. Recorded variables included trial identifiers (e.g., NCT or EudraCT numbers), drug regimen, dosing, route of administration, patient population characteristics, therapeutic exposure status (treatment-naïve versus treatment-exposed), ADA frequency, neutralizing antibody data, timepoints of ADA assessment, and assay characteristics such as format, sensitivity, and drug tolerance when reported.

One of the primary challenges in curating the IDC DS V1 was the varied measurements and interpretations used to report ADA outcomes. Terms such as total, treatment-emergent, transient, persistent, and treatment-boosted ADA were reported inconsistently across sources. To harmonize this, only two frequency types were retained or derived where possible: (1) treatment-naïve (i.e., all patients ADA-positive in unexposed cohorts) or baseline (i.e., all patients ADA-positive at baseline), and (2) treatment-emergent (including both treatment-induced and treatment-boosted rates).

The term “ADA frequency” is used instead of “ADA incidence” in this dataset to denote the percentage of patients who tested positive for ADAs at a given timepoint. ADA incidence, as defined by Shankar et al. (2014), refers to the number of patients who developed treatment-emergent ADA (that is, treatment-induced or treatment-boosted) during a study (27). Providing an accurate measure for incidence requires individual patient-level information, including baseline and follow-up ADA status. However, most data sources provided only summary-level data in the form of ADA positivity at discrete time points without individual patient profiles or baseline status. This limitation necessitated the use of ADA frequency as a more general and descriptive metric to the proportion of ADA-positive patients. ADA frequency is then interpreted in the context of therapeutic exposure. In therapeutic-naïve patients, frequency denotes any ADA positivity at baseline or in placebo or comparator cohorts at a given timepoint. In therapeutic-exposed patients, ADA frequency is interpreted as treatment-emergent ADA at a given timepoint.

### 2.3 T cell epitope prediction

Potential immunogenic CD4^+^ T cell epitopes were predicted computationally using NetMHCIIpan-4.3 EL (28) for nine common HLA-DR alleles: HLA-DRB1*01:01, HLA-DRB1*03:01, HLA-DRB1*04:01, HLA-DRB1*07:01, HLA-DRB1*08:01, HLA-DRB1*09:01, HLA-DRB1*11:01, HLA-DRB1*13:01, and HLA-DRB1*15:01. These alleles were selected based on their broad global population coverage (85.61%) and the availability of extensive MAPPs training data (29–31). Peptides ranging from 12 to 20 amino acids were considered to be strongly presented on HLA-DR if the predicted binding core (a nine-mer peptide) had a percentile rank score within the top 10%. To filter likely tolerized epitopes, the predicted binding cores were queried against the natural antibody repertoire in the Observed Antibody Space (OAS) using the BioPhi humanness analysis tool (32, 33). Binding cores found in more than 110 human OAS subjects were excluded from further analysis. Binding cores were also removed if their amino acid sequences matched nine-mer peptides from the human reference proteome (34). In addition, cores overlapping with ‘knobs-into-holes’ mutations were excluded, as such mutations have been suggested to have a lower risk of immunogenicity (35, 36). The remaining binding cores were retained as potentially immunogenic CD4^+^ T cell epitopes.

### 2.4 Statistical analysis

The association between anti-drug antibody (ADA) frequency and individual immunogenicity risk factors was investigated. Initial analysis revealed that ADA frequencies significantly deviated from a normal distribution. Consequently, we employed a non-parametric bootstrapping approach to determine if clinical risk factors impacted ADA frequency. Specifically, a 1,000-fold resampling procedure was performed at the cohort level, in which ADA frequencies within each clinical factor category were sampled with replacement and group medians recalculated to generate an empirical distribution of outcomes. Observed group medians (or median differences relative to a reference group) were then compared against these bootstrap distributions, and two-sided p-values were estimated as the proportion of replicates yielding medians more extreme than the observed value. This framework not only avoids assumptions of normality but also enables flexible hypothesis testing, such as assessing whether the median ADA frequency of each group significantly differs from the overall median or from a designated reference group. When comparisons against a designated reference group were performed, the reference category was selected *a priori* as a representative baseline condition for the variable under investigation (e.g., absence of co-medication, the most neutral route of administration, or a non-immunomodulatory therapeutic category). The specific reference group used for each analysis is indicated in the corresponding figure legend. The factors evaluated included those recorded in the Clinical Trial table: sampling time (from the Trial Arm Timepoint Description column), dose (from the Dosing Description column), dosing interval (from the Therapeutic Dosing Schedule Description column), route of administration (from the Therapeutic Route of Administration column), and co-medication (from the Co-administered drugs column). Additionally, the drug mechanism of action (MOA) (from the Therapeutics table, Mechanism of Action column) was evaluated. Monotonic relationships between ADA frequency and the continuous numerical factors (sampling time, dose, and dosing interval) were not assumed; therefore, these variables were converted into categorical variables by binning them into quintiles (five equal groups) for subsequent analysis. For the number of predicted T-cell epitopes, we hypothesized a monotonic, linear relationship with ADA frequency and therefore employed standard linear regression to assess this association.

To assess the combined effect of multiple variables, we performed a multivariate logistic regression. For this analysis, ADA frequency was first dichotomized into a binary outcome (low ADA vs. high ADA) using a 10% frequency cutoff. This transformation makes the analysis more robust by mitigating the influence of extreme outliers in ADA frequencies, which could otherwise disproportionately affect the regression model. The binary ADA category was then regressed against the following risk factors: drug MOA, indication, number of T-cell epitopes, dosing interval, route of administration, co-medication, dose level, and study year.

The relative importance of each risk factor was quantified by its contribution to the model’s total deviance. Deviance is a measure of model fit; a larger reduction in deviance upon adding a specific factor to the model indicates a more significant contribution of that factor to explaining the ADA outcome.

## 3 Results

### 3.1 Creating a fit-for-purpose data architecture

The IDC database architecture was built using TIDY data principles (37) and refined through multiple rounds of pilot testing and expert feedback. While the current schema was designed to enable integration of heterogeneous public data sources, future iterations will aim to further align with established biomedical ontologies and controlled vocabularies to enhance interoperability and facilitate integration with external datasets. The architecture consists of three relational data tables, Therapeutics, Sequences, and Clinical Trials, connected by shared unique identifiers which are outlined below. The IDC DS V1 was designed as an initial demonstration dataset intended to validate the data architecture and illustrate potential applications of the framework, rather than an exhaustive catalog of all published clinical immunogenicity observations.

The Therapeutics table (**Supplementary Table 1**) assigns an IDC unique Therapeutic ID (e.g. PR_0001) to each drug and captures a range of high-level attributes spanning molecule features, targets, mechanism of action, regulatory status, and biosimilar-to-originator relationships. Information was aggregated from various sources including FDA and EMA product labels, WHO INN databases (38), and curated drug repositories such as DrugBank (23) and AdisInsight (39). External identifiers, including INNs, trade names, and the drugs manufacturer. The IDC DS V1 reflects information up to the end of 2024.

The Sequences table (**Supplementary Table 2**) provides amino acid sequences organized by chain or domain, providing an IDC unique Sequence ID (e.g. SQ_0001) and linking to a parental IDC Therapeutic ID. Sequence verification was attempted by confirming identity in at least two independent sources, including IMGT (25), KEGG (40), Drugs@NCATS (41), Thera-SabDab (24), or directly in patents. The sequence source information is provided for traceability and when amino acid sequences could not be verified, entries were flagged accordingly under the “Sequence Verified?” column in the Therapeutic table.

The Clinical Trials table (**Supplementary Table 3**) captures trial- and cohort-level features with respective unique IDC identifiers, a Trial ID for each unique clinical trial and an IDC Row identifier for each unique trial-cohort-timepoint and/or ADA frequency readout combination. External clinical study identifiers are provided under the “Trial External Source” and “External Source Identifier” columns (e.g. clinicaltrials.gov and NCT00611208) and provide a persistent identifier originating from public registries. It catalogs treatment regimens, patient cohort characteristics, assay properties, ADA/neutralizing ADA (nADA) frequencies, assessment timepoints, and a variety of other features and metrics. Reported ADA measures were unified into an ADA frequency at the assessment timepoint (see **Supplementary Information** for further details). While ADA incidence requires individual-level longitudinal data, this dataset uses ADA frequency as a practical alternative to the available summary-level data most commonly reported in ClinicalTrials.gov, FDA labels, and the EU Clinical Trials Register.

Together, these components establish IDC DS V1 (**Supplementary Table 4**), a framework for standardized immunogenicity data capture and enable side-by-side exploration of clinical, mechanistic, and molecular features. Please refer to the “Variables Explained” tab for further descriptions for each field.

### 3.2 Clinical immunogenicity data set overview

**Figure 1:**
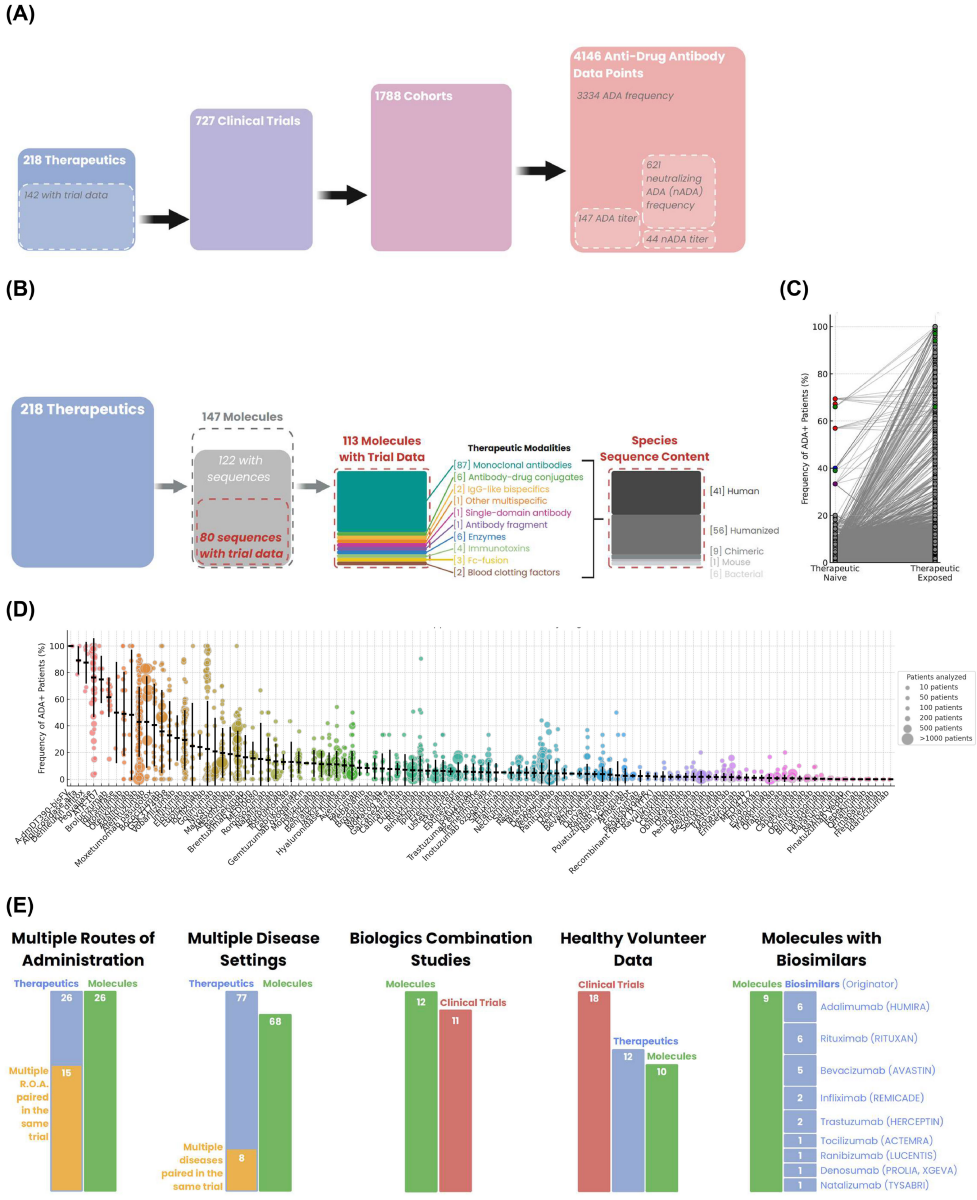
An overview of the clinical immunogenicity dataset. **(A)** The dataset features 218 therapeutics with 142 having clinical trial data. In total, the database integrates 1,788 cohorts across 727 clinical trials, yielding 4,146 ADA-related datapoints: 3,334 ADA frequency, 621 nADA frequency, 147 ADA titer, and 44 nADA titer measurements. **(B)** Of the 218 therapeutics captured, there are 148 unique molecules with 122 amino acid sequences identified for those molecules. Eighty amino acid sequences have clinical trial data captured. A variety of protein modalities and source species are represented amongst the 113 molecules having clinical trial data captured, the vast majority representing monoclonal antibodies. **(C, D)** Reported ADA+ patient frequency for each datapoint is shown. **(C)** Datapoints are separated based on labeling as coming from patients not exposed to a therapeutic (Therapeutic Naive) or those exposed to a therapeutic (Therapeutic Exposed). Red = Brolucizumab; Blue = Otelixizumab; Purple = Utomilumab, Green = Denileukin Difitox. **(D)** Datapoints are grouped and colored by therapeutic. The size of each datapoint is proportional to the number of patients assessed for ADAs within the patient cohort at that specific timepoint. Mean ADA+ frequency and standard deviation are shown. **(E)** The number of therapeutics, molecules and clinical trials captured within the dataset are shown for each of the given context.

The IDC DS V1 features 218 therapeutics (e.g., drug products) with 142 for which clinical trial data was available (**Figure 1A**). In total, the dataset integrates 1,788 cohorts across 727 clinical trials, yielding 4,146 ADA-related datapoints: 3,334 ADA frequency, 621 nADA frequency, 147 ADA titer, and 44 nADA titer measurements. This scale enables comparative analyses across therapeutic classes and clinical contexts.

The composition of molecules (e.g., unique proteins) captured in the dataset is summarized in **Figure 1B**. Monoclonal antibodies predominate (n=87), with several antibody–drug conjugates (n=6), bispecific or multispecific antibodies (n=3 total), single-domain/fragment antibodies (n=2), enzymes (n=6), immunotoxins (n=4), Fc-fusion proteins (n=3), and blood factors (n=2). Of the 147 molecules captured, 122 had amino acid sequences available and 80 of those have clinical trial data captured in the dataset. The species origin for molecules captured skews toward humanized (n=56) and human (n=41), with smaller counts of chimeric (n=9), mouse (n=1), and bacterially derived proteins (n=6).

Immunogenicity datapoints were labeled as originating from either therapeutic-naïve or therapeutic-exposed cohorts. While most ADA frequencies increase at timepoints post exposure, several molecules (Brolucizumab, Otelixizumab, Utomilumab, and Denileukin Diftitox) exhibited elevated pre-existing ADA in naïve patients at baseline (**Figure 1C**). Unless otherwise noted, all subsequent analyses were focused on therapeutic exposed populations only.

The therapeutics captured in the Clinical Trials table cover a wide range of ADA frequencies and illustrate the variability of ADA frequencies reported for the same therapeutic across trials, cohorts and timepoints (**Figure 1D**). Fluctuations in reported ADA rates may reflect multiple contributing factors, thus these findings underscore the importance of interpreting immunogenicity data within the specific clinical context and conditions under which data were obtained. To track immunogenicity rates at multiple levels, we also provide aggregated ADA frequencies by cohort (**Supplementary Figure S2A**), trial (**Supplementary Figure S2B**), therapeutic (**Supplementary Figure S2C**), and molecule (**Supplementary Figure S2D**), with data provided in **Supplementary Table 5**.

Finally, the data set provides opportunities to examine key features of great interest to the field (**Figure 1E**). Examples include 26 therapeutics with multiple routes of administration (ROA) (15 trials with multiple ROA in the same trial), 77 used within multiple indications (8 within the same trial), 11 used in combination, 18 studies in healthy volunteers, and 9 molecules with biosimilars. These groupings define the analytic space for some representative evaluations presented in the next section and highlight opportunities for the immunogenicity community to further expand the dataset.

### 3.3 Insights into drivers of immunogenicity risk

Substantial variability in anti-drug antibody (ADA) frequency was observed across clinical cohorts in the IDC dataset, highlighting both inter- and intra-therapeutic heterogeneity (**Supplementary Figure S2A**). Some therapeutics demonstrated consistently high ADA frequencies across trials (e.g., Denileukin Diftitox), while others exhibited broad variability, potentially reflecting trial- and cohort-specific factors (e.g., Adalimumab, Infliximab). At scale, these differences raise fundamental questions: What drives ADA frequency variation across biologics? Which drug-intrinsic and drug-extrinsic features matter most? To begin addressing these, we evaluated the impact of several hypothesized drivers of clinical immunogenicity using both univariate patterns and case-specific examples.

**Figure 2:**
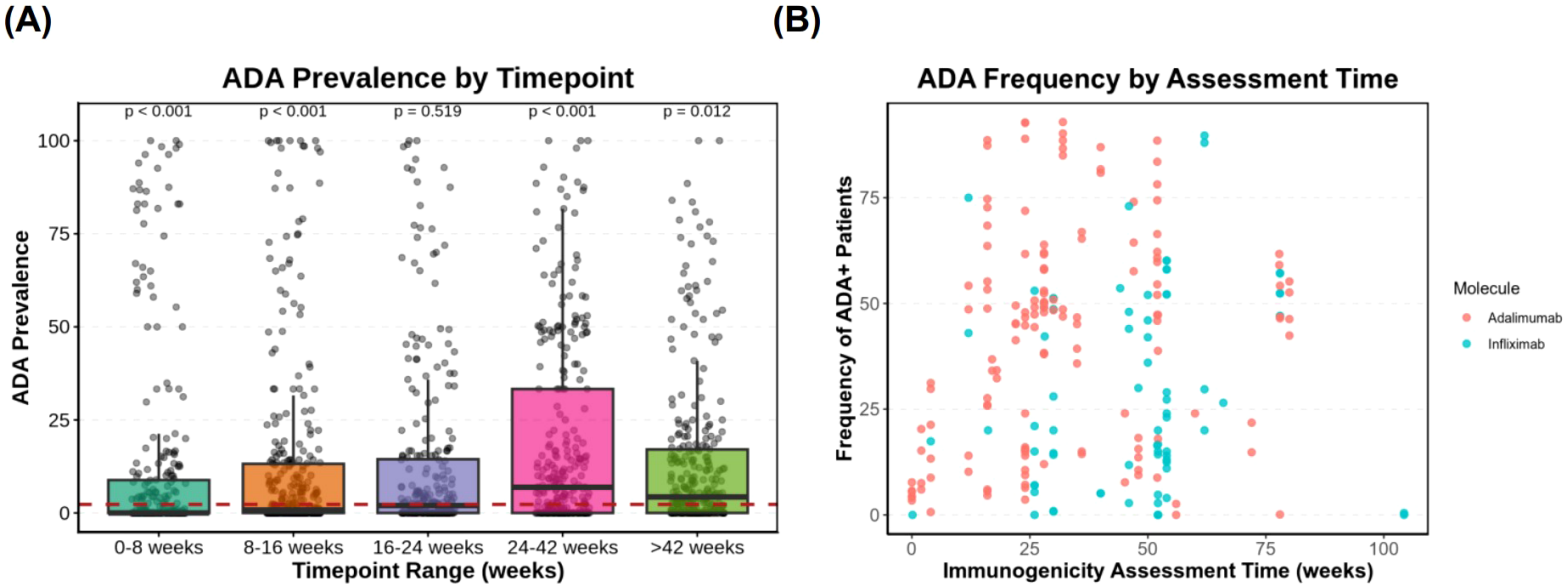
ADA frequency measurements over time. **(A)** Distribution of ADA frequencies across clinical cohorts plotted by weeks since first therapeutic exposure. Each point represents a cohort-level measurement at a reported timepoint. 1000-fold bootstrapping was used to test if the median of each group is significantly different from the overall median. **(B)** Examples from Adalimumab and Infliximab clinical trials showing ADA frequencies measured at multiple timepoints, with values plotted relative to weeks post first dose.

ADA sampling schedules captured in the dataset exhibit an expectedly wide range, with some cohorts assessed within a week of dosing and others up to several years later. ADA frequency generally increased over time, peaking around 24–42 weeks after the first dose (**Figure 2A**). Early elevated ADA frequencies may reflect prior exposure (e.g. common vaccination against diphtheria toxin and its in immunotoxin Denileukin Diftitox (42) or in rare cases cross-reactive pre-existing ADAs (e.g. shared epitopes between common pathogenic microbes and Brolucizumab (43)(**Figure 1C**). Analyses of Adalimumab and Infliximab (**Figure 2B**) further illustrate this trend, with both therapeutics demonstrating rising ADA frequencies in later post-dose samples and peaking at 25–50 weeks. These observations reinforce the impact sampling time has on the reported frequency of ADA within a cohort. For this reason, we generated additional data tables listing the maximum ADA frequency observed for a cohort within a trial (cohort level), the average ADA frequency of all cohorts within a trial (trial level) and the average ADA frequencies calculated by aggregating all ADA positive and negative patients amongst all cohorts for a particular drug (therapeutic level) or protein (molecule level) (**Supplementary Table 5**).

### 3.4 Immunogenicity and the role of therapeutic context

**Figure 3:**
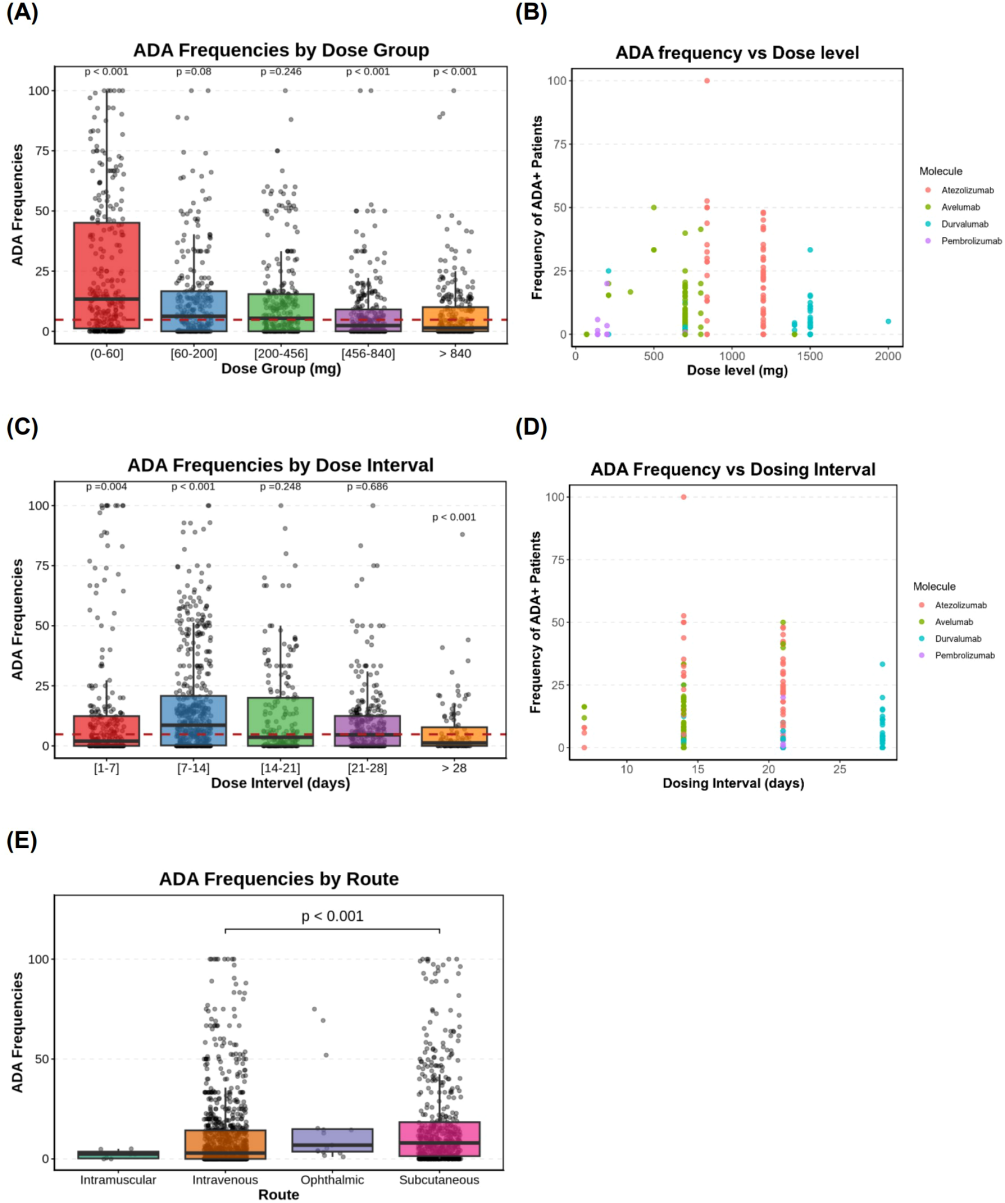
Impacts of dose amount, dose interval and route of administration on ADA frequency. **(A)** ADA frequencies across cohorts grouped by dose amount categories. Each point represents a cohort-level measurement at a reported timepoint. A 1000-fold bootstrapping approach was used to test whether the median of each group differs significantly from the overall median. **(B)** ADA frequencies for Durvalumab, Atezolizumab, Avelumab, and Pembrolizumab cohorts, stratified by dose amount. Each point represents a cohort-level measurement at a reported timepoint. **(C)** ADA frequencies across cohorts grouped by dosing interval categories. Each point represents a cohort-level measurement at a reported timepoint. A 1000-fold bootstrapping approach was used to test whether the median of each group differs significantly from the overall median. **(D)** ADA frequencies for Durvalumab, Atezolizumab, Avelumab, and Pembrolizumab cohorts, stratified by dosing interval. Each point represents a cohort-level measurement at a reported timepoint. **(E)** ADA frequencies across cohorts stratified by route of administration (subcutaneous vs. intravenous). Each point represents a cohort-level measurement at a reported timepoint. A 1000-fold bootstrapping approach was used to test whether the median differs significantly between subcutaneous and intravenous routes.

The clinical setting in which a therapeutic is delivered (e.g. the dose amount, dosing interval and ROA) can potentially impact ADA induction rates. Across the dataset, higher ADA frequencies were observed at lower dose levels, with the highest seen for >0.2–60 mg per dose (**Figure 3A**). These findings may reflect lower immune tolerance thresholds at subtherapeutic exposure or potential assay interference due to higher drug levels at sampling. However, when evaluating a smaller set of therapeutics all of which target the PD-1/PD-L1 pathway in oncology settings (Durvalumab, Atezolizumab, Avelumab, Pembrolizumab), higher ADA frequencies were observed at intermediate dosing levels (700–1200 mg) (**Figure 3B**). Previous reviews evaluating the immunogenicity of these kinds of immunotherapics had not determined a dose-dependent effect on nADA incidence, however this was likely due to limited available data (44).

Regarding dose interval, we observed a peak in ADA frequency for regimens administered at approximately 7–14 day intervals (**Figure 3C**), a pattern reminiscent of standard prime-boost immunization schedules. A similar trend was observed for the smaller set of anti-PD-1/PD-L1 therapeutics at 14- and 21-day intervals (**Figure 3D**), suggesting that immune system re-exposure timing may modulate the likelihood of ADA formation. The ROA may also impact immunogenicity, with subcutaneous (SC) administration classically thought to be more immunogenic than intravenous (IV) due to enhanced uptake by antigen-presenting cells in peripheral tissues (14). In the IDC DS V1, SC routes were associated with modestly higher ADA frequencies than IV (**Figure 3E**). There are currently only 3 intramuscular and 4 ophthalmic administered therapeutics in the IDC DS V1, limiting the insights which can be gained for those ROA. Only a small subset of therapeutics had datapoints associated with both SC and IV administration, with an even smaller subset providing comparative data within the same clinical trial. While patients receiving SC administered therapeutics broadly exhibited higher ADA frequencies compared to those given IV treatments; when ROA were compared within the same trial minimal differences were observed (**Supplementary Figure S3**). Specifically, in the four identified trials where both IV and SC were performed (NCT01435382 for Bococizumab, NCT01089725 for Tanezumab, NCT00638989 for Tralokinumab and NCT00950300 and NCT01401166 for Trastuzumab) at most a 5% difference was observed between ROAs. Together, these findings suggest that ROA may represent a contributing factor to ADA risk, however, additional within-trial comparisons are required to conduct more robust evaluations and draw definitive conclusions.

### 3.5 Patient immune status is key

**Figure 4:**
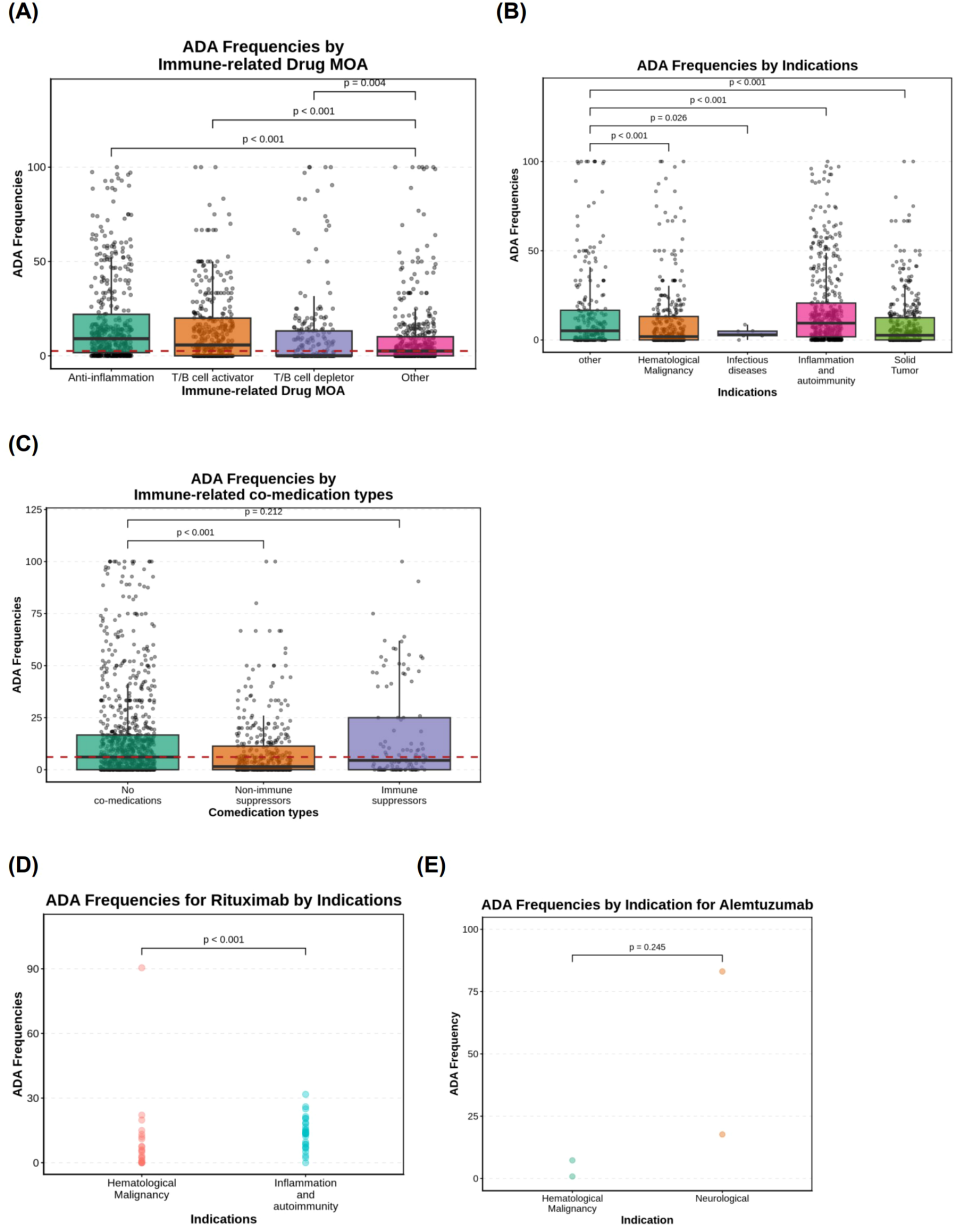
ADA frequencies observed across various drug and comedication mechanisms of action and disease indications. **(A)** ADA frequencies across biologics grouped by immune mechanism of action. Each point represents a cohort-level measurement at a reported timepoint. The “other” group was used as the baseline reference, and a 1000-fold bootstrapping approach was applied to test whether the median of each group differs significantly from the baseline. **(B)** ADA frequencies across patient cohorts grouped by disease indication. Each point represents a cohort-level measurement at a reported timepoint. The “other” group was used as the baseline reference, and a 1000-fold bootstrapping approach was applied to test whether the median of each group differs significantly from the baseline. **(C)** ADA frequencies across cohorts stratified by comedication type. Each point represents a cohort-level measurement at a reported timepoint. The “no-comedications” group was used as the baseline reference, and a 1000-fold bootstrapping approach was applied to test whether the median of each group differs significantly from the baseline. **(D, E)** ADA frequencies for Rituximab **(D)** and Alemtuzumab **(E)** cohorts stratified by disease indication. Each point represents a cohort-level measurement at a reported timepoint. Wilcox tests were used to calculate the p values in **(D, E)**.

A biologic’s mechanism of action (MOA) can influence its immunogenicity both directly via immune system modulation, and indirectly based on the pre-existing immune status of the patient population in which it is used (21). When grouped by immunomodulatory profile, biologics with T and B cell activating MOAs (e.g. checkpoint inhibitors) had higher average ADA frequencies than those with T and B cell depleting or immunologically neutral MOAs (**Figure 4A**). Biologics with T and B cell depleting MOAs had the lowest overall median ADA frequencies, significantly lower than all other MOA groups including “Other” (**Figure 4A**). While therapeutics having anti-inflammatory MOAs had the highest overall ADA frequencies (**Figure 4A**), this may be confounded by the heightened immune status of the patient population or increased antigen presenting cell uptake due to expression of the target receptor. Indeed, patients with inflammatory or autoimmune conditions exhibited the highest ADA frequencies compared to other disease indication cohorts (**Figure 4B**).

Concomitant medications frequently differ between patient populations and can significantly impact immune status and ADA responses. When evaluated in aggregate, cohorts labeled as having received immunosuppressive agents (e.g., methotrexate or corticosteroids) or no comedications exhibited higher average ADA frequencies than cohorts treated with non-immune suppressive co-medications (**Figure 4C**). While this finding contradict previous reports (45), these co-medications are often utilized in autoimmune patients who exhibit increased ADA frequencies (**Figure 4B**). When evaluated on a per therapeutic basis the inclusion of suppressive co-medications most often (4 of 5 therapeutics) led to reduced ADA frequencies (**Supplementary Figure S4**). However, no apparent differences were observed for the very few datapoints with cohorts evaluated within the same trial and thus inclusion of additional intratrial evaluations of co-medications remain a priority for future iterations of the database.

To further evaluate the impact of patient cohort immune status on ADA frequency, we compared two molecules used in different disease settings: Rituximab (an anti-CD20 murine-human chimeric monoclonal antibody that depletes B cells) and Alemtuzumab (an anti-CD52 humanized monoclonal antibody that depletes T and B cells). In the dataset these biologics exhibited higher ADA rates in autoimmune compared to oncology patient cohorts (**Figures 4D, E**). This observation further emphasizes the need to interpret immunogenicity endpoints in the context of all relevant clinical features.

### 3.6 T cell epitope content and sequence based risks

**Figure 5:**
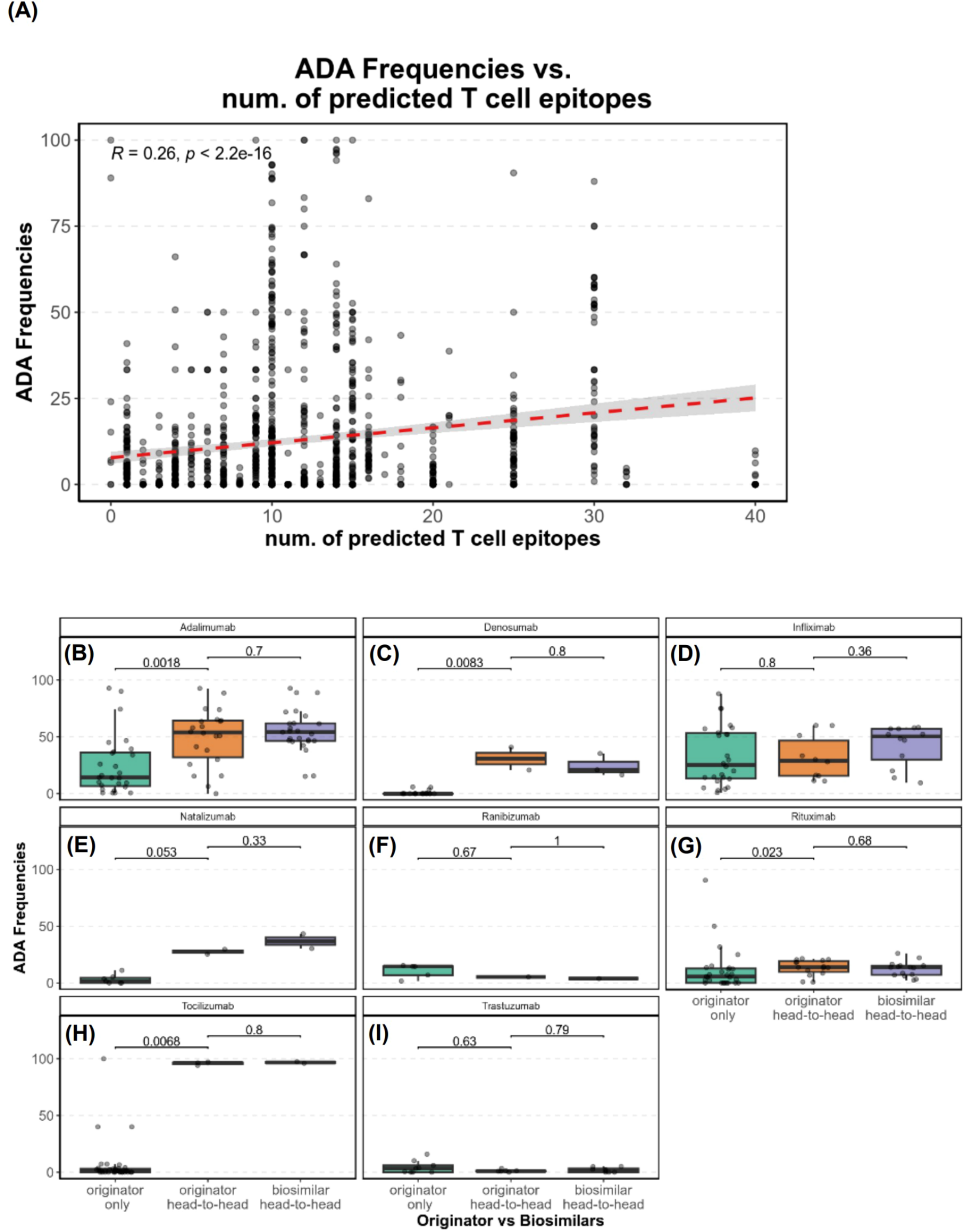
Correlation of T cell epitope content on ADA frequencies and comparison of sequence identical biosimilars. **(A)** Relationship between predicted CD4^+^ T cell epitope counts and ADA frequencies across therapeutics. Each point represents a therapeutic-level measurement. Linear regression was used for testing the association. **(B–I)** ADA frequencies reported for an originator drug evaluated in a trial on its own (originator only) and originator–biosimilar pairs evaluated in the same clinical trial (head-to-head). Each point represents a cohort-level measurement at a reported timepoint. t tests were used to compare ADA frequencies between originators and biosimilars.

Intrinsic sequence features also represent a major risk factor for developing unwanted immunogenicity. One such feature involves the quantity and quality of T cell epitopes contained within the biologic’s amino acid sequence. Using an industry standard peptide presentation model (NetMHCIIpan-4.X (30)), we enumerated the total number of predicted MHC class II presented peptides containing non-germline encoded residues (e.g. predicted CD4+ T cell epitope load) for each therapeutic (see Methods) and compared it to cohort-level ADA frequencies (**Figure 5A**). Higher predicted CD4+ T cell epitope counts were weakly but positively correlated with ADA rates, consistent with prior reports utilizing a similar methodology (46). These results suggest that epitope-based modeling can provide useful predictions of clinical immunogenicity but provide an incomplete perspective when evaluated in isolation. Additional variables such as differences in protein structure, post-translational modifications, host cell protein content (47), and other drug product-related features may also modulate the immunogenic response, however these variables fall outside the scope of the current dataset.

Biosimilars share the same primary amino acid sequence with the originator or reference product and are required to demonstrate no clinically meaningful differences in safety, purity, or potency (48). Any differences in ADA frequency observed between biosimilars and reference products would thus likely stem from minor variations in CQAs, process-related impurities, formulation or manufacturing differences, bioanalytical assay design, or trial context. Across seven originator–biosimilar pairs in the IDC DS V1, ADA frequencies were generally comparable when both products were evaluated within the same trial (**Figures 5B–I**). We also observe that originators typically reported lower ADA frequencies during their initial clinical development, which may reflect historical assay formats (49) or differences in patient selection. These observations are in line with regulatory trends towards reducing potentially unnecessary clinical measures during evaluation of biosimilar products with historically minimal immunogenicity liabilities (50) and underscore the value of organizations publicly reporting immunogenicity metrics.

### 3.7 Quantifying variable contribution through multivariate analysis

**Figure 6:**
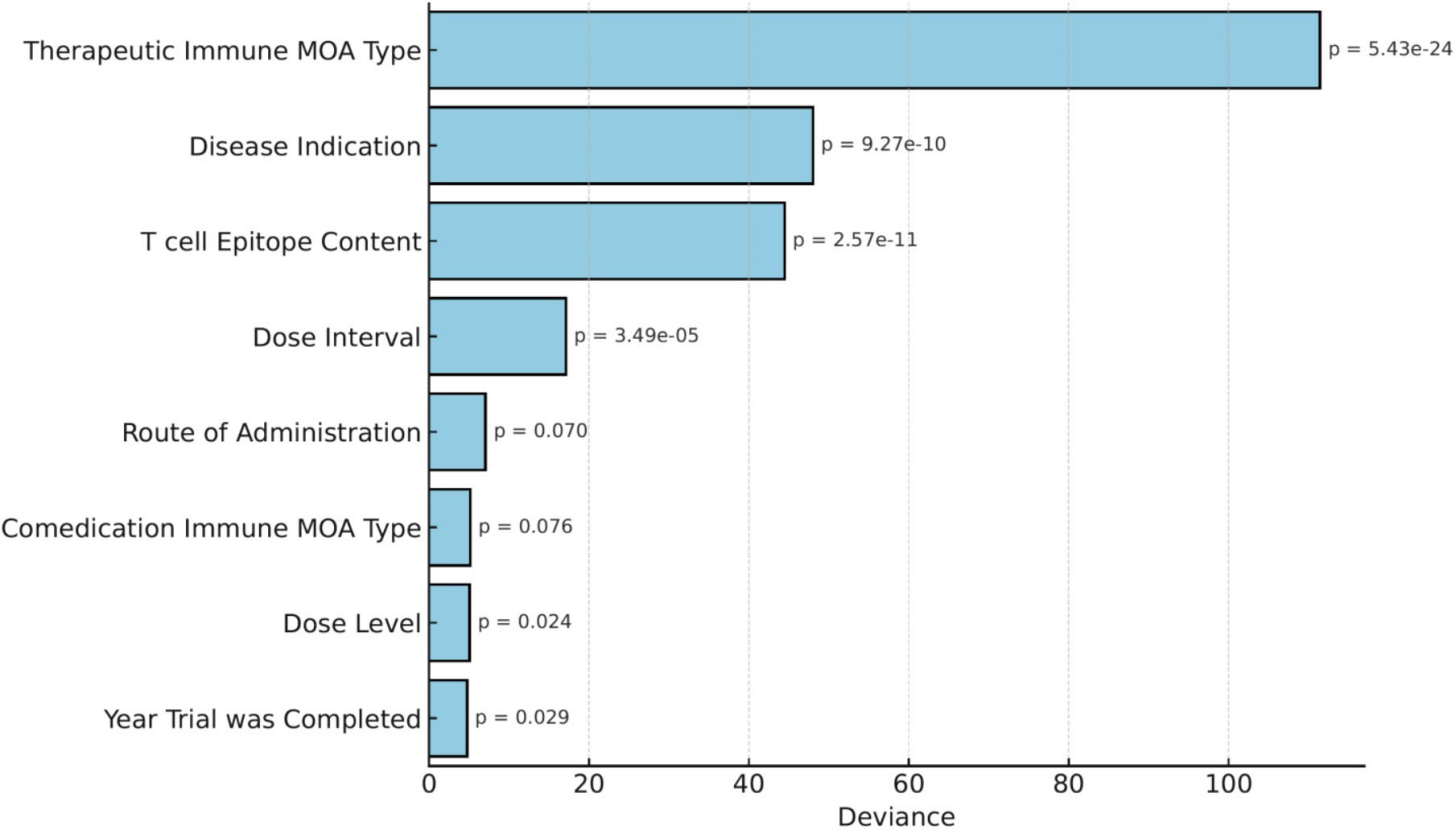
Multivariate analysis for drivers of immunogenicity risk. Multivariate logistic regression of cohort-level ADA frequencies dichotomized into low (<10%) and high (≥10%) categories. Variables included drug mechanism of action, disease indication, predicted T cell epitope content, dosing interval, route of administration, comedication type, dose level, and trial year. The relative importance of each factor was quantified by its contribution to the reduction of model deviance.

To assess the relative contribution of each variable while accounting for confounders, we performed multivariate regression using a range of clinical and therapeutic features captured in the dataset. As shown in **Figure 6** and **Supplementary Table 6**, the top three variables associated with higher ADA frequency were therapeutic MOA, disease indication and predicted T cell epitope content. Conversely, variables such as ROA, co-medication immunomodulation type and dose level had weaker or inconsistent associations similar to the year in which the trial was completed. These analyses highlight the multifactorial nature of immunogenicity and support current integrative risk assessment practices that combine clinical, mechanistic, and sequence-based inputs.

## 4 Discussion

The IDC was established to provide a standardized, publicly accessible dataset linking therapeutic, sequence, and clinical features to clinical immunogenicity outcomes. This first release integrates over one hundred therapeutics and thousands of cohorts, capturing ADA frequencies at multiple levels of aggregation. The dataset offers flexible architecture for interoperability and future expansion. Its scale and diversity enable systematic evaluation of immunogenicity risk factors, while also underscoring the multifactorial and context-dependent nature of ADA development.

Insights from the IDC DS V1 both confirm and extend prior observations in the field. SC administration is often considered more immunogenic than IV (51). Across the dataset, SC delivery was associated with modestly higher ADA rates (**Figure 3E**), but significant differences were observed for only 4 of the 24 therapeutics having data from both ROA. This pattern mirrors Davis et al., who reported increased risk for 6 of 20 therapeutics, and Felderman et al., who found no significant correlation when controlling for confounders (52, 53). These findings indicate that ROA may contribute to immunogenicity but is rarely a dominant driver, and more paired SC/IV data, ideally from the same trials, are needed for robust conclusions.

Patient immune context could influence ADA outcomes. Therapeutic MOA, disease indication, and co-medications often overlap, complicating attribution of individual effects. Immunosuppressive concomitant medications are likely to influence ADA frequencies, however, outcomes in our dataset were variable as trials in this dataset were skewed towards autoimmune and oncological indications. Biologics with anti-inflammatory MOAs are predominantly tested in autoimmune settings, where underlying immune status differs substantially from that of oncology cohorts. Case studies such as Rituximab and Alemtuzumab illustrate this point (**Figure 4D, E**), underscoring the importance of contextual interpretation when considering immunogenicity risk across different indications and regimens.

Sequence features remain key determinants of immunogenicity. Consistent with similar, recent analyses (46), predicted CD4+ T cell epitope load showed a weak but significant correlation with ADA frequency via univariate analyses (**Figure 5A**) and was highlighted as a major contributor via multivariate analyses (**Figure 6**). Biosimilars, which are expected to share the same primary amino acid sequence as their reference products, exhibited comparable ADA rates when tested within the same trials, demonstrating a high degree of consistency when a significant subset of factors is controlled for (**Figures 5B–I**). Differences between early originator trials and later biosimilar studies likely reflect historical variation in assay format, sensitivity, cut-off determination and patient selection. Greater availability of detailed assay characteristics would substantially improve understanding across the field. Sequence-level comparisons beyond biosimilar relationships, such as assessing sequence similarity across unrelated biologics, may provide additional insight into shared immunogenic determinants. Systematic evaluation of such relationships, particularly when combined with controlling for non-sequence-based features impacting immunogenicity risk (such as mechanism of action, co-medications, etc.) represents a promising avenue for future investigation.

MHC II presentation prediction and the identification of MHC binding cores utilized a relatively inclusive threshold of ≤10% percentile rank for elution score for predicted peptide presentation without weighting by predicted binding affinity, number of alleles predicted per peptide or allele prevalence. More advanced approaches that incorporate quantitative features such as percentile rank, promiscuity of presentation on multiple HLAs, HLA allele frequency, or consideration of experimentally validated epitopes (e.g., from resources such as the Immune Epitope Database (IEDB) (www.iedb.org)) could enhance the interpretability and predictive power of sequence-derived features. However, integration of such datasets should involve careful curation to account for variability in experimental validation for reported epitopes and experimentation to determine optimal weighting schemas for each risk feature. Additionally, immune tolerance prediction was incorporated into the potential CD4^+^ T cell epitope calculations presented here by employing filtering strategies based on sequence identity to the human proteome and measured human antibody amino acid sequences from the Observed Antibody Space database (33). While this approach is consistent with common practices in the field, self or human sequence identity alone does not guarantee immunological tolerance, particularly in the context of autoimmune disease or altered immune states. Future analyses incorporating more nuanced considerations for protein amino acid sequences likely driving central and peripheral tolerance, as well as patient-specific immune context, may further improve the predictive value of sequence-based risk assessments.

In this study we utilize predicted CD4^+^ T cell epitope content as a sequence-derived feature, while B cell epitope prediction tools were not included. This reflects current limitations in the field, as B cell epitope prediction methods remain less mature and are often optimized for tasks such as identifying antibody-binding sites or mapping epitopes for known antibody–antigen pairs, rather than providing quantitative metrics directly applicable to immunogenicity risk assessment (54). As such tools continue to improve, datasets such as IDC DS can serve as valuable references to evaluate and optimize model predictions.

Multivariate regression highlighted four leading variables associated with ADA risk: therapeutic MOA, disease indication, predicted epitope content, and dosing interval (**Figure 6**). The top three were also identified in the same order by Zicheng et al. in a smaller but more controlled dataset, providing independent validation (46). In contrast, route of administration, comedication type, and dose level showed weaker or inconsistent associations.

Several limitations of the IDC DS V1 warrant acknowledgement. ADA outcomes were harmonized to a single baseline or treatment-emergent frequency per cohort-timepoint combination (see Methods), enabling comparability but excluding alternative measures such as transient, persistent or total ADA incidence. Single, aggregate therapeutic- or molecule-level values are provided for convenience (**Supplementary Table 5**) but should not be used in isolation, as they obscure heterogeneity arising from trial design and cohort context. ADA assay parameters (including sensitivity, drug tolerance, and antigen source in the case of biosimilar trials) were infrequently reported, limiting the depth of interpretation and the ability to assess the quality of the ADA data reported. Reported ADA frequencies can evolve as clinical development progresses, with larger, more diverse populations and updated assay technologies often providing higher sensitivity and drug tolerance. In the absence of assay drug tolerance and pharmacokinetic measures, it is not possible to exclude potential impacts of drug exposure (e.g. serum drug concentrations) on reported ADA measures. Biosimilarity and interchangeability studies can also report frequencies that may differ from early trials. Thus, utilization of trial start and end dates can provide longitudinal perspective on reported ADA frequencies. Finally, nADA frequencies, ADA titers, and clinical outcomes such as efficacy or safety impacts hold substantial value for understanding immunogenicity risk, however these datapoints were infrequently reported, minimizing meaningful conclusions that can be drawn beyond total ADA frequency. Despite these caveats, the IDC DS V1 represents a significant advancement over prior published datasets (19–22), integrating a broad set of molecules, clinical contexts, and mechanistic features into a unified framework. The dataset supports both large-scale comparative analyses and targeted case studies, facilitating identification of consistent immunogenicity drivers while also illustrating the limitations of single-factor interpretations.

To improve accessibility and support long-term sustainability, the IDC initiative will maintain a public website (www.immunogenicitydb.org) and repository (github.com/Immunogenicity-Database-Collaborative/IDC-DB) that serve as the primary locations for dataset distribution, documentation, updates, and future community engagement activities. These resources provide a foundation for ongoing expansion of the database and facilitate broader participation from the immunogenicity community.

Currently planned future efforts will improve and extend the database across several dimensions, including data breadth, depth, structure and standardization. To support this evolution, we have initiated coordinated community efforts toward building the IDC DS V2 with an initial three-pronged approach. First, expansion of clinical data through automated data acquisition pipelines and API-based integration with resources such as ClinicalTrials.gov and other registries. We plan to broaden clinical data coverage by targeting additional biosimilars, non-antibody modalities (e.g., peptides, Fc-fusions, nanobodies, enzymes) and enriching for trials that capture features of interest such as multiple routes of administration, immunomodulatory concomitant medications, and the same therapeutics evaluated in multiple disease indications. Second, we hope to capture more detailed clinical data and metadata, particularly indicators of ADA impact (such as safety, efficacy and pharmacokinetics) and features that enable better assessment of data quality (such as sensitivity and drug tolerance). Third, we seek to expand the publicly available IDC Database by aggregating and harmonizing published non-clinical datasets through development of standardized data structures for capturing preclinical immunogenicity risk readouts. Such initial datasets could include *in vitro* immune cell assays (dendritic cell uptake, MHC II-associated peptide proteomics, T cell antigenicity, etc.) and relevant biophysical properties (aggregation propensity, polyreactivity, post-translational modifications).

An important parallel objective is the continued advancement of data standardization, interoperability, and alignment with FAIR (Findable, Accessible, Interoperable, Reusable) data principles. While the current data structure was designed as a fit-for-purpose framework to integrate heterogeneous public data sources, future iterations will incorporate more formal mappings to established ontologies and controlled vocabularies. Examples include linking biologic species origin to NCBI taxonomy identifiers or humanness assessment metrics, standardizing molecular targets using resources such as the Protein Ontology or UniProt, and aligning disease indications with structured vocabularies such as the Disease Ontology. These efforts will improve consistency, enable more robust cross-dataset comparisons, and facilitate integration with complementary biomedical resources.

More broadly, the IDC DS highlights the need for improved standardization in how immunogenicity data are generated, reported, and shared. Key variables such as ADA assay characteristics and reported immunogenicity endpoints are often inconsistently described across studies, limiting context-aware comparisons. We believe the IDC framework, developed by domain experts in immunogenicity risk assessment, represents a meaningful step toward structured data capture in this field, while recognizing that it is not yet fully optimized and will continue to evolve. In this context, the IDC DS is positioned not only as a data resource, but also as a platform to help inform emerging best practices and promote greater consistency across studies.

A key longer-term objective is to enable contribution of unpublished datasets from the broader community, including data from programs with significant immunogenicity-related challenges that are currently underrepresented in public sources. We anticipate that a structured and extensible framework such as IDC DS will help lower barriers to data sharing and support more collaborative, data-driven progress in understanding and mitigating immunogenicity risk. Challenges in harmonization of datasets will remain, but industry-wide collaborative efforts offer a path forward, with approaches such as federated learning providing an avenue to leverage sensitive datasets without compromising data privacy (55).

The release of the IDC DS V1 showcases both the current state of the data architecture and the constraints of available public data. By contributing data and feedback, the field can ensure that IDC database evolves into a durable shared resource for identifying predictors of immunogenicity, benchmarking risk assessment methods, and improving the safe and effective use of biologics.

## Data Availability

The entirety of the Immunogenicity Database Collaborative Dataset (IDC DS) V1 is openly licensed via CC BY 4.0 (creativecommons.org/licenses/by/4.0/), provided in **Supplementary Table 4** and made accessible through the IDC website (www.immunogenicitydb.org) and public GitHub repository (https://github.com/Immunogenicity-Database-Collaborative/IDC-DB). Future database releases, documentation updates, and community contribution information will be maintained through these resources.
