# Supplementary material for: The immunogenicity database collaborative: a standardized, publicly available database for clinical immunogenicity observations and insights
Source: Front Immunol. 2026 Jun 30;17:1816949. doi: 10.3389/fimmu.2026.1816949 (PMC13365037; doi:10.3389/fimmu.2026.1816949)
Supplement: Supplementary file 1 [file DataSheet1.pdf]

Supplementary Material

1 Supplementary Methods

1.1 Dataset architecture, construction and quality control further explained

In addition to the Therapeutics, Sequences and Clinical Trials tables, two supporting tables were provided: Variables Explained and Controlled Language. The Variables Explained table provided definitions for each variable across the three main datasets, while the Controlled Language table standardized data entry by restricting certain fields to predefined dropdown options. For example, the field “Therapeutic Exposure Status” was limited to two controlled terms: Therapeutic Exposed and Therapeutic Naive. The purpose of this was to explicitly distinguish undosed subjects so that those receiving placebo or a different drug in the trial were not incorrectly included in the result.

Each biologic is indexed using a unique Therapeutic ID, allowing consistent traceability across therapeutic attributes, sequence-level features, and clinical trial outcomes. This relational framework enabled users to explore ADA frequency in the context of therapeutic modality, molecular characteristics, and trial-specific variables.

1.2 Generating aggregate datapoints at the cohort, clinical trial, therapeutic and molecule level

Supplementary Table S5 provides an aggregated ADA frequency table across four hierarchical levels: cohort, trial, therapeutic, and molecular. This table was generated by using the clinical trial dataset but with columns capturing ADA frequency estimates and corresponding identifiers for each aggregation level. At the cohort level, a unique cohort_group_id was assigned to each trial arm–exposure–drug combination, with the ADA frequency (cohort_ADA) defined as the maximum frequency of ADA-positive patients observed at any time point (max_ADA_time) within that cohort, and the corresponding number of patients (N_at_max_ADA) noted. Trial-level frequencies were calculated using a weighted average of cohort_ADA values for all cohorts within a trial_group_id (defined as trial–exposure–drug combinations), weighted by N_at_max_ADA. The total number of patients contributing to each trial-level frequency is indicated as N_of_trial_ADA. At the patient level, we report two forms of aggregate frequency: therapeutic and molecular. Therapeutic patient-level ADA frequency (PRID_ADA) was computed for each protein-exposure grouping (PR_group_id) as a weighted average of cohort-level ADA frequencies. Similarly, molecule-level ADA frequency (INN_ADA) was computed for each INN-exposure grouping (INN_group_id). The sample size used for each weighted average is reported as N_of_PRID_ADA and N_of_INN_ADA, respectively. The aggregated values did allow for comparisons across trials and molecules; however, increasing the level of aggregation obscured meaningful variation introduced by study-specific factors such as dose, regimen, or population heterogeneity.

2 Supplementary Figures and Tables

(A) (B)

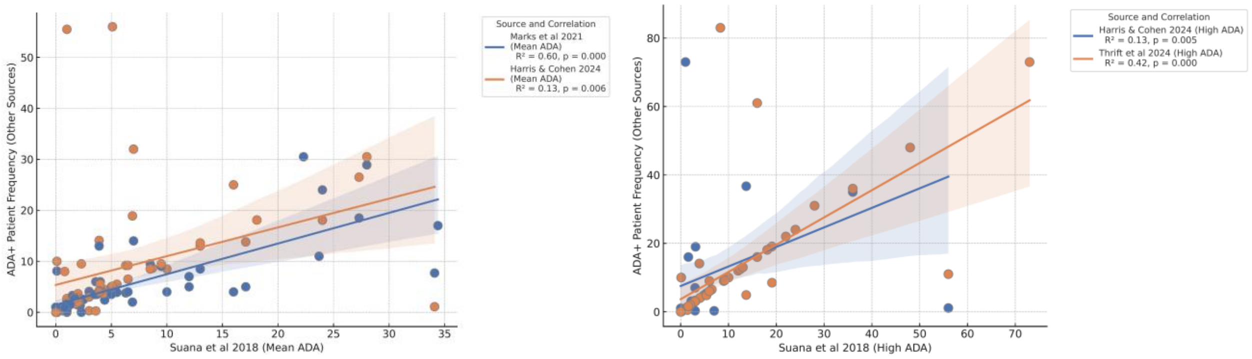

Figure S1: ADA frequencies reported by commonly referenced datasets

Reported ADA rates for therapeutics from commonly referenced clinical immunogenicity data table sources are compared. Suana et al 2019 = Sauna et al. Trends Biotechnol. 2018 Oct;36(10):1068-1084. doi: 10.1016/j.tibtech.2018.05.008; Marks et al 2021 = Marks et al. Bioinformatics. 2021;37(22):4041-4047. doi:10.1093/bioinformatics/btab434; Harris & Cohen 2024 = Harris CT, Cohen S. BioDrugs. 2024 2024;38(2):205-226. doi:10.1007/s40259-023-00641-2; Thrift et al 2024 = Thrift et al. Briefings in Bioinformatics. 2024;25(3)doi:10.1093/bib/bbae123. (a) Mean ADA rates reported by the indicated sources for any therapeutics shared between sources. Mean ADA frequency were either provided or calculated by taking the reported low and high ADA frequencies for each therapeutic in the data tables. (b) High ADA frequencies reported by the indicated sources for any therapeutics shared between sources. Pearson correlation best fit lines and statistics are provided.

**INN Name**	Adalimumab	Adalimumab
**Therapeutic ID**	PR_0003	PR_0004
**Trade name**	HUMIRA	HYRIMOZ
**Manufacturer**	AbbVie	Sandoz
**Labelled as Biosimilar?**	No	Yes
**Progeny of**		PR_0003
**Protein Description**	Human monoclonal antibody (IgG1) against TNFa	Human monoclonal antibody (IgG1) against TNFa
**Protein Modality**	Monoclonal antibody	Monoclonal antibody
**Species**	Human	Human
**Antibody Backbone**	human IgG1	human IgG1
**Light Chain**	kappa	kappa
**Fc Modifications**	None	None
**Conjugate Modification**	Unconjugated	Unconjugated
**Conjugate Description**	None	None
**Expression System**	Chinese hamster ovary (CHO) cells	Chinese hamster ovary (CHO) cells
**Sequence IDC database Identifier(s)**	SQ_0003, SQ_0004	SQ_0003, SQ_0004
**Sequence Verified?**	Yes	Yes
**Target(s) (Protein/Molecule)**	TNFa	TNFa
**Mechanism of Action**	Inhibition of TNFa binding to TNFa receptor	Inhibition of TNFa binding to TNFa receptor
**Furthest Development Stage Reached**	Approval	Approval
**FDA approved**	Yes	Yes
**First FDA approval**	2002	2018
**EU approval**	Yes	
**First EU approval**	2016	

Table S1: Overview of the therapeutics data table

Sequence ID	SQ_0003	SQ_0004
Parental Molecule Therapeutic ID	PR_0003	PR_0003
INN Name	Adalimumab	Adalimumab
Chain Multiplicity	2	2
Chain Identifier	A	B
Chain Descriptor	Heavy Chain 1	Light Chain 1
Amino Acid Sequence	EVQLVESGGGLVQPGRSLRLSCAASGFTFDDYAMHWVRQAPGKGLEWVSAITWNSGHIDYADSVEGRFTISRDNAKNSLYLQMNSLRAEDTAVYYCAKVSYLSTASSLDYWGQGTLVTVSSASTKGPSVFPLAPSSKSTSGGTAALGCLVKDYFPEPVTVSWNSGALTSGVHTFPAVLQSSGLYSLSSVVTVPSSSLGTQTYICNVNHKPSNTKVDKKVEPKSCDKTHTCPPCPAPELLGGPSVFLFPPKPKDTLMISRTPEVTCVVVDVSHEDPEVKFNWYVDGVEVHNAKTKPREEQYNSTYRVVSVLTVLHQDWLNGKEYKCKVSNKALPAPIEKTISKAKGQPREPQVYTLPPSRDELTKNQVSLTCLVKGFYPSDIAVEWESNGQPENNYKTTPPVLDSDGSFFLYSKLTVDKSRWQQGNVFSCSVMHEALHNHYTQKSLSLSPGK	DIQMTQSPSSLSASVGDRVTITCRASQGIRNYLAWYQQKPGKAPKLLIYAASTLQSGVPSRFSGSGSGTDFTLTISSLQPEDVATYYCQRYNRAPYTFGQGTKVEIKRTVAAPSVFIFPPSDEQLKSGTASVVCLLNNFYPREAKVQWKVDNALQSGNSQESVTEQDSKDSTYSLSSTLTLSKADYEKHKVYACEVTHQGLSSPVTKSFNRGEC
Sequence Source	https://opig.stats.ox.ac.uk/webapps/sabdab-sabpred/therasabdab...	https://opig.stats.ox.ac.uk/webapps/sabdab-sabpred/therasabdab...

Table S2: Overview of the sequences data table

**Trial ID**	CT0601
**Trial External Source**	Clinicaltrials.gov
**External Source Identifier**	NCT04230213
**Molecule Assessed for ADA INN Name**	Adalimumab
**Therapeutic Assessed for ADA Trade name**	HUMIRA
**Therapeutic Assessed for ADA ID**	PR_0003
**IDC Row identifier**	CT0601_A1_001
**Immunogenicity testing**	Anti-Adalimumab (Amsparity) Antibodies
**Therapeutic Exposure Status**	Therapeutic Exposed
**Trial Arm Description**	Switching arm: humira/adalimumab (amsparity)
**Trial Arm Timepoint Description**	Tp2: week 10 to week 16
**Dosing Description**	Participants after completing tp1, were randomized to receive adalimumab (amsparity) 40 mg once every 2 weeks subcutaneously for 6 weeks during treatment period 2 (tp2)
**Therapeutic Dosing Schedule Description**	40 mg adalimumab (amsparity) once q2w sc from week 10 to 16
**Disease Indication Category**	Inflammation and autoimmunity
**Disease Indication Description**	Rheumatoid Arthritis
**Co-administered drugs**	Methotrexate
**Patient Population**	Diagnosis of RA based on 2010 ACR/EULAR for RA for at least a 4 month duration. Moderately to severely active RA based on local standard of care
**Trial Start Date**	1/13/2020
**Trial End Date**	6/22/2021
**Therapeutic Route of Administration**	Subcutaneous
**Immunogenicity Assessment Reported Up To (Days)**	112
**Number of Patients analyzed for ADA**	213
**Number of ADA+ patients**	55
**Frequency of ADA+ patients**	25.8
**ADA detection assay used**	Serum samples were analyzed using a validated electrochemoluminescent (ECL) immunoassay for ADA assessment. Samples positive for ADA were further tested for neutralizing activity using a validated cell based NAb assay
**ADA assay sensitivity or calculation**	ADA positive was defined as ADA titer >=1.88 while NAb positive was defined as NAb titer >=0.70
**Number of patients analyzed for ADA titers**	211
**Average ADA titers**	0.83
**nADAs detected**	Yes
**Number of patients analyzed for nADAs titers**	55
**Average nADA's titers**	0.712
**Number of patients analyzed for nADA**	55
**Number of patients with nADAs**	22
**Frequency nADA+ patients reported**	40
**Frequency of patients with hypersensitivity or injection site reactions**	Not Reported
**ADA interpreted to impact PK**	Not Reported
**ADA interpreted to impact Efficacy**	Not Reported

Table S3: Overview of the clinical trials data table

(A)

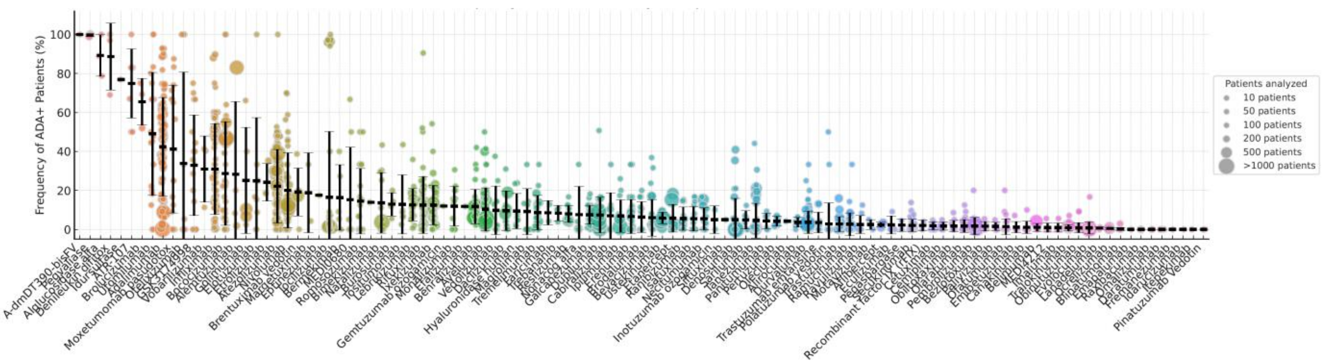
Frequency of ADA+ Patients (%)
Patients analyzed
10 patients
50 patients
100 patients
200 patients
500 patients
>1000 patients

(B)

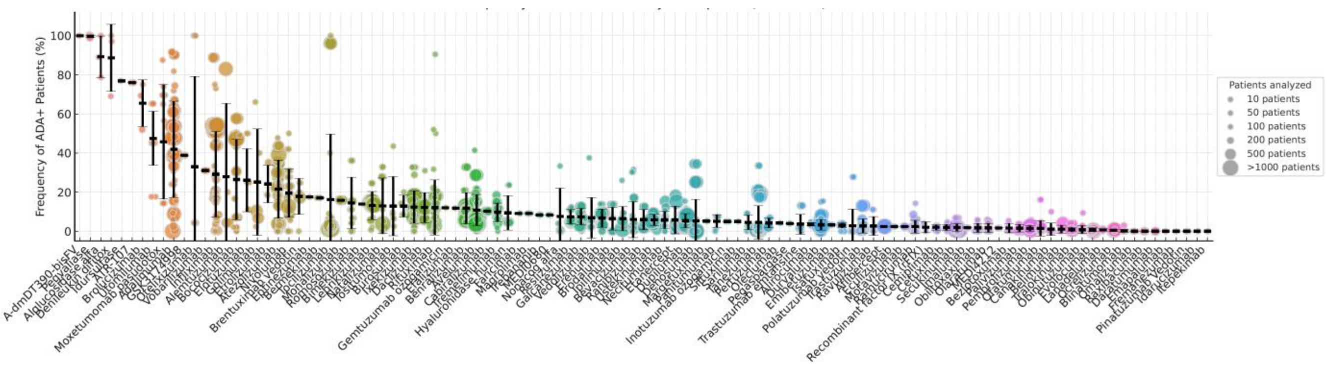
Frequency of ADA+ Patients (%)
Patients analyzed
10 patients
50 patients
100 patients
200 patients
500 patients
>1000 patients

(C)

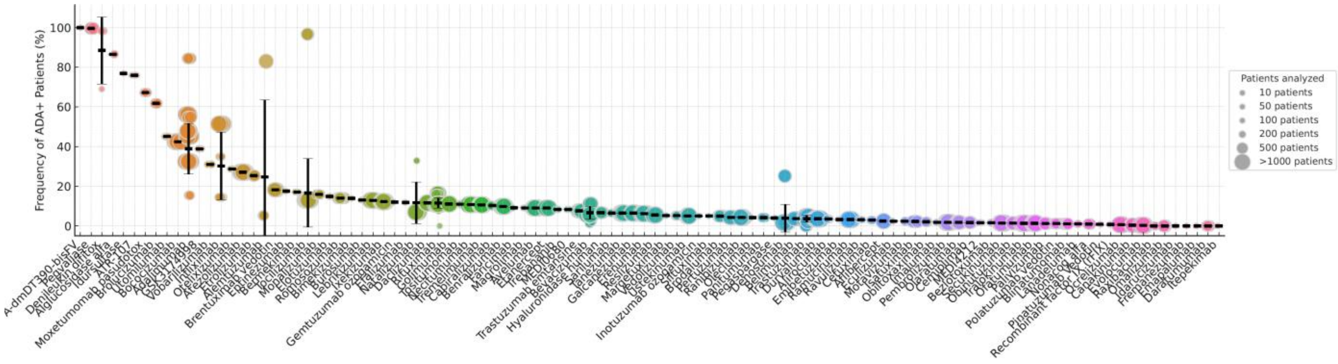
Frequency of ADA+ Patients (%)
Patients analyzed
10 patients
50 patients
100 patients
200 patients
500 patients
>1000 patients

(D)

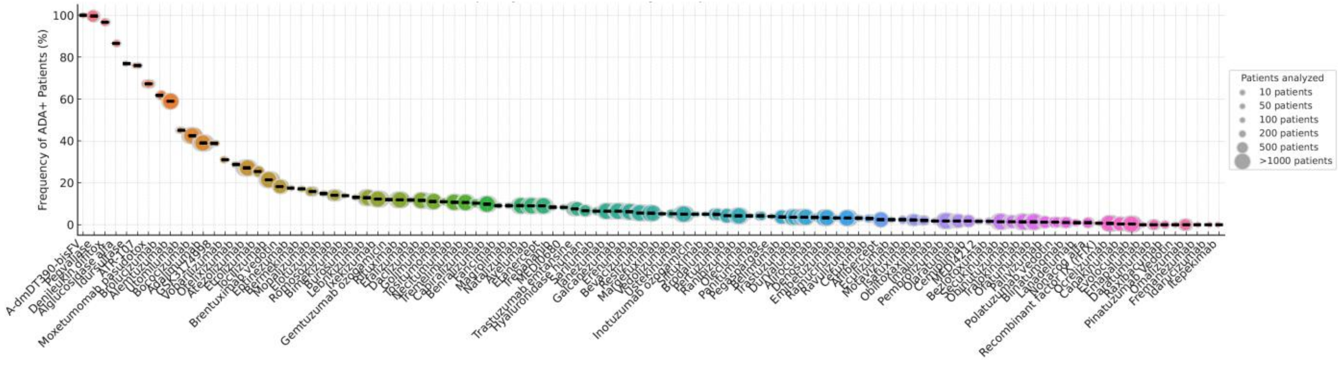
Frequency of ADA+ Patients (%)
Patients analyzed
10 patients
50 patients
100 patients
200 patients
500 patients
>1000 patients

Figure S2: ADA Frequencies Captured in the IDC DS V1 Across All Therapeutics

(a) Cohort level ADA frequencies. Maximum observed ADA frequency is shown for each therapeutic across all clinical trial cohorts. Each data point represents a unique cohort, defined by a distinct trial-arm–exposure–drug combination (cohort_group_id, e.g., PR_0127_Therapeutic Naïve_CT0001_A1) in Table S5. ADA frequency (cohort_ADA) reflects the highest frequency of ADA-positive patients observed at any time point within the cohort. The corresponding time point is indicated by max_ADA_time, and the number of patients tested at that time point is given by N_at_max_ADA. Circle size denotes the number of patients analyzed per cohort. (b) Trial level ADA frequencies. Each point represents the trial-level ADA frequency for a therapeutic protein, derived by aggregating cohort-level data across all relevant trial arms sharing a common trial–exposure–drug grouping (trial_group_id) as seen in Table S5. ADA frequency (trial_ADA) was computed as a weighted average of maximum cohort-level ADA values, with weights corresponding to the number of patients tested at the peak time point (N_at_max_ADA) within each cohort. Circle sizes reflect the total number of patients contributing to each trial-level estimate (N_of_trial_ADA). (c) Therapeutic level ADA frequencies. Each data point represents the ADA frequency aggregated across all clinical trial cohorts associated with that therapeutic and exposure status. ADA frequency (PRID_ADA) was calculated as a weighted average of maximum cohort-level ADA frequencies (cohort_ADA), using the number of patients tested at the time of maximum ADA (N_at_max_ADA) within each cohort as weights. The protein-exposure grouping is defined by PR_group_id, and the total number of patients contributing to each value is indicated as N_of_PRID_ADA. Circle sizes reflect the patient count per therapeutic grouping. (d) Molecule level ADA frequencies. Each data point represents the ADA frequency for a therapeutic molecule, aggregated across all trials and cohorts associated with that molecule and exposure status. ADA frequency (INN_ADA) was calculated as a weighted average of maximum cohort-level ADA frequencies (cohort_ADA), using the number of ADA-tested patients at the time of maximum ADA (N_at_max_ADA) within each cohort as weights. Molecule groupings are defined by INN_group_id, and the number of patients contributing to each estimate is indicated by N_of_INN_ADA. Circle sizes correspond to the patient sample size per molecule.

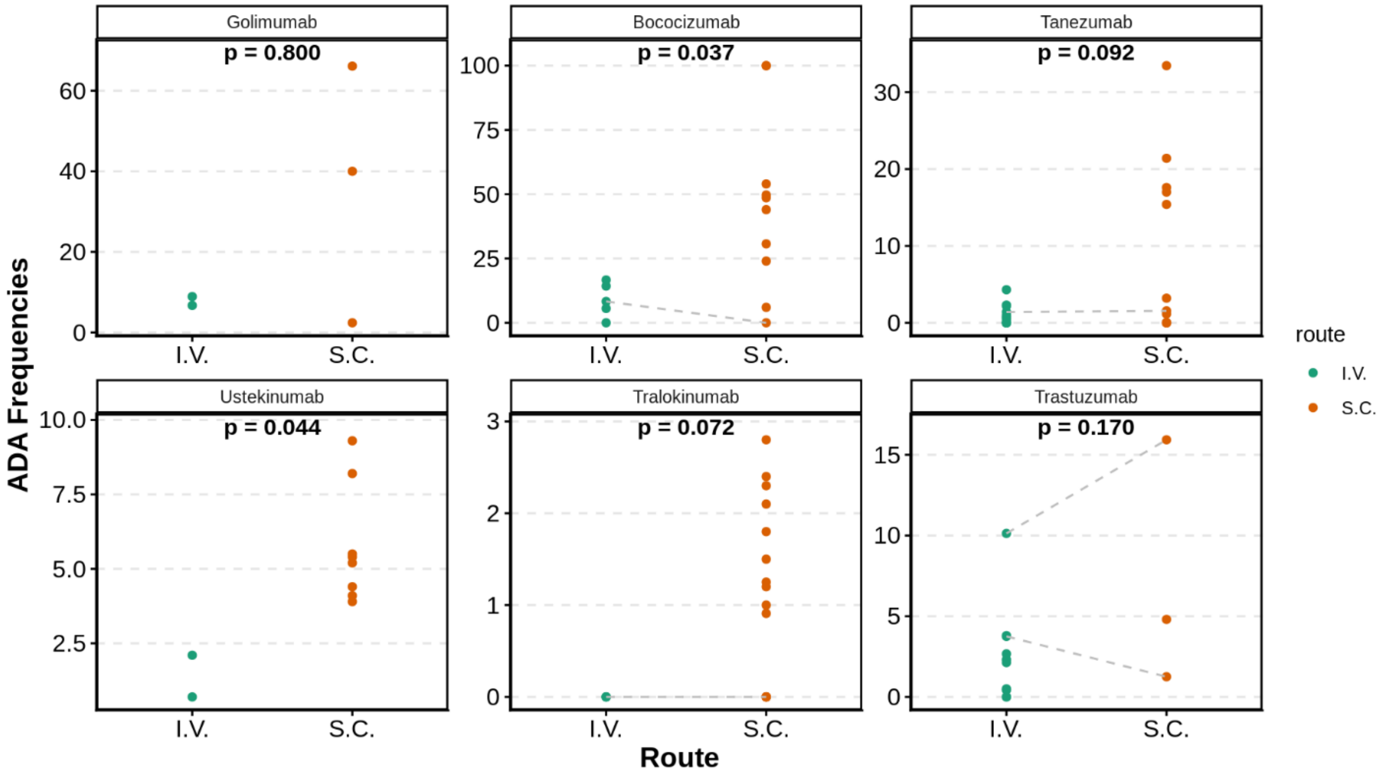

Figure S3: ADA Frequency Comparison for Therapeutics Evaluated by Both Subcutaneous (SC) and Intravenous (IV) Administration. ADA frequencies were compared for therapeutics with reported immunogenicity data for both SC and IV administration in the IDC DS V1. Each point represents a cohort-level ADA frequency measurement at a reported assessment timepoint. Therapeutics are plotted individually to facilitate within-molecule comparison and reduce confounding from differences in therapeutic modality, target biology, or mechanism of action. Wilcox tests were used to calculate the p values comparing reported ADA frequencies between the IV and SC treated patient cohorts. Dotted lines connecting cohorts between routes of administration indicate examples where both IV and SC were evaluated within the same trial.

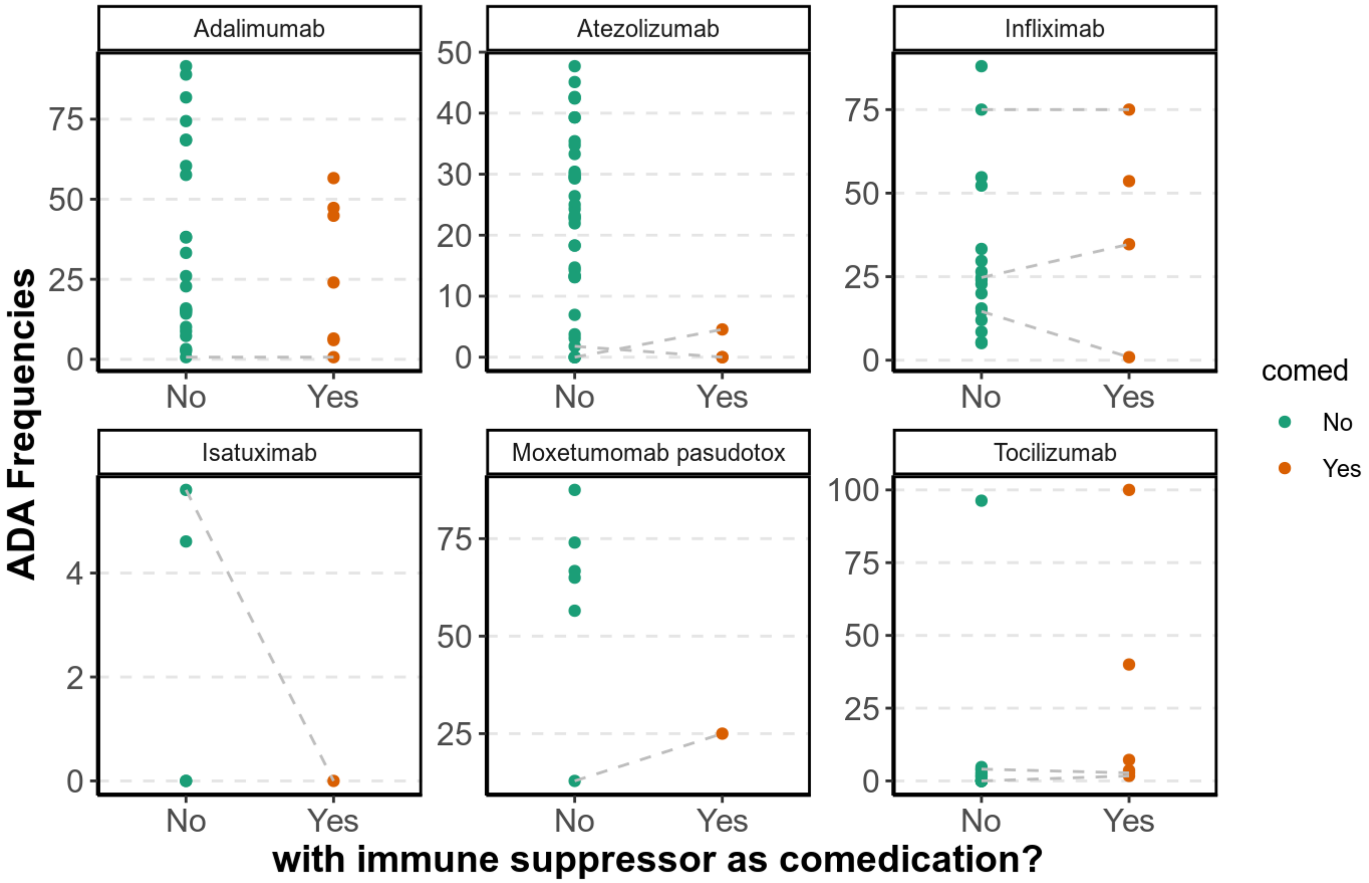

Figure S4: ADA Frequencies for Biologics Evaluated in Patient Cohorts Reported to be Utilizing Immune Suppressive Co-medications. ADA frequencies are shown for therapeutics evaluated in patient populations reported with and without immune-suppressive co-medications in the IDC DS V1. Each point represents a cohort-level ADA frequency measurement at a reported assessment timepoint. Therapeutics are plotted individually to facilitate comparison across molecules while preserving the influence of co-medication status within the clinical context in which ADA responses were measured. Cohorts were grouped according to whether they received immune suppressive co-administered treatments. Dotted lines connecting cohorts indicate examples where the same biologic was evaluated both in the presence and absence of immune-suppressive co-medications.

**Variable**	**Df**	**Deviance**	**Resid. Df**	**Resid. Dev**	**Pr(>Chi)**
Therapeutic Immune MOA Type	3	111.4186	1207	1378.433	5.43E-24
Disease Indication	4	48.03786	1210	1489.851	9.27E-10
T cell Epitope Content	1	44.47694	1214	1537.889	2.57E-11
Dose Interval	1	17.13003	1203	1351.075	3.49E-05
Route of Administration	3	7.050262	1199	1339.265	0.070313
Comedication Immune MOA Type	2	5.154917	1205	1373.278	0.075967
Dose Level	1	5.072558	1204	1368.205	0.024307
Year Trial was Completed	1	4.759851	1202	1346.315	0.029131

Table S6: Multivariate Regression Results for Features Associated with High ADA Frequency
